# Institutional Needs

**Published:** 1914-12-19

**Authors:** 


### Institutional Needs.

### THE "SANITAS" SPRAY.

Now that civilians—in fact, the whole nation—are awake to the problems of sickness and disease because of the war, "Sanitas Fluid" as a deodorant and antiseptic is of special interest. Since all advertised disinfectants do not disinfect, the public will note not merely "Sanitas" as a fluid for washing purposes, but its use in purifying the air of sick rooms by the spray, especially in connection with the oxygen contained in it.

### SEMI-PORCELAIN HOSPITAL WARE.

It is necessary that hospital ware should be strong, with a good surface, and impervious, and for this purpose the "Perfection" ware made by Grimwades, Ltd., Stoke-on-Trent, is worth attention. Typical products of the firm, whose wares are made of white semi-porcelain, are the "Handy" female urinal and child's bed-pan, the "Perfection" pus or vomiting basins, the back wall of which is twice as high as the front, which are made in three sizes, and the "Perfection" bed and douche pan. The "Perfection" male urinal, which can be used in conjunction with the "Perfection" bed-pan, is designed so as not to require holding when in position, and is thus useful in cases of incontinence. The illustration of the two, shown in conjunction, which has appeared in our business columns, makes this distinguishing feature fully clear: in all the bed-pans particular care is taken to prevent by the curve of the design any pressure upon the spine. On the other hand there are no tortuous curves to make these shapes difficult to cleanse or empty, and the "Perfection" dressing-basins are planned both for ear and eye work. Grimwades' designs are well thought out, and managers requiring a new stock of hospital ware could not do better than consult their catalogue and price-list.

